# Transcriptome disclosure of hormones inducing stigma exsertion in *Nicotiana tabacum* by corolla shortening

**DOI:** 10.1186/s12864-024-10195-4

**Published:** 2024-03-28

**Authors:** Xiaoquan Zhang, Juxu Li, Xuexia Xing, Hongchen Li, Songtao Zhang, Jianbo Chang, Fengjie Wei, Yongfeng Zhang, Jinhui Huang, Xuelin Zhang, Zhaojun Wang

**Affiliations:** 1 College of Tobacco Science, Henan Agricultural University, 450046 Zhengzhou, China; 2 Henan Provincial Branch of China National Tobacco Corporation, 450018 Zhengzhou, China; 3 Sanmenxia Branch of Henan Provincial Tobacco Corporation, 472000 Sanmenxia, China; 4 Shangluo Branch of Shanxi provincial Tobacco Company, 726000 Shangluo, China; 5 College of Agronomy, Henan Agricultural University, 450046 Zhengzhou, China

**Keywords:** Stigma exsertion, *Nicotiana tabacum*, Plant hormones, Corolla, RNA-seq analysis

## Abstract

**Background:**

Stigma exsertion is an essential agricultural trait that can promote cross-pollination to improve hybrid seed production efficiency. However, the molecular mechanism controlling stigma exsertion remains unknown.

**Results:**

In this study, the *Nicotiana tabacum* cv. K326 and its two homonuclear-heteroplasmic lines, MSK326 (male-sterile) and MSK326SE (male-sterile and stigma exserted), were used to investigate the mechanism of tobacco stigma exsertion. A comparison of the flowers between the three lines showed that the stigma exsertion of MSK326SE was mainly due to corolla shortening. Therefore, the corollas of the three lines were sampled and presented for RNA-seq analysis, which found 338 candidate genes that may cause corolla shortening. These genes were equally expressed in K326 and MSK326, but differentially expressed in MSK326SE. Among these 338 genes, 15 were involved in hormone synthesis or signal transduction pathways. Consistently, the content of auxin, dihydrozeatin, gibberellin, and jasmonic acid was significantly decreased in the MSK326SE corolla, whereas abscisic acid levels were significantly increased. Additionally, seven genes involved in cell division, cell cycle, or cell expansion were identified. Protein-protein interaction network analysis identified 45 nodes and 79 protein interactions, and the largest module contained 20 nodes and 52 protein interactions, mainly involved in the hormone signal transduction and pathogen defensive pathways. Furthermore, a putative hub gene coding a serine/threonine-protein kinase was identified for the network.

**Conclusions:**

Our results suggest that hormones may play a key role in regulating tobacco stigma exsertion induced by corolla shortening.

**Supplementary Information:**

The online version contains supplementary material available at 10.1186/s12864-024-10195-4.

## Background

Stigma exsertion is an agricultural trait in which the stigmas in the flower buds stretch out of the corolla before anthesis, and the stamens remain wrapped by the corolla and fail to self-pollinate normally. This trait can be used to improve hybrid seed production efficiency and the application of heterosis in crop breeding [1, 2].

Stigma exsertion is caused by changes in the relative lengths of different organs during flower development. Wild tomato species bear exserted stigmas because their styles are significantly longer than those of cultivated tomato species, and this difference is due to variations in style cell length [3, 4]. Similarly, the cultivated tomato line T431 also exhibits exserted stigmas, but this is attributed to an increased number of style cells [5]. High-temperature-induced stigma exsertion in cultivated tomatoes is caused by the stamen shortening more severely than the pistil [6, 7].

Several critical regulatory factors and quantitative trait loci (QTLs) that regulate stigma exsertion have been identified in various plant species, including tomato [3, 6], rice [8], wheat [9], and *Arabidopsis thaliana* [10, 11]. In tomato, a QTL designated as *stigma exsertion 2.1* (*se2.1*) has been mapped to chromosome two. This QTL contains at least five closely related genes, including three controlling stamen length, one controlling anther dehiscence, and one controlling style length [4]. Of these five genes, *Style2.1* is considered to be the primary contributor to stigma exsertion [3].

Unlike that in tomato, stigma exsertion in rice is considered a quantitative trait controlled by multiple genes [8]. Nine QTLs for stigma exsertion frequency have been detected in the recombinant inbred lines derived from a cross between the *japonica* rice variety *Asominori* and *indica* rice variety IR24 [12]. Twenty-three genomic loci significantly associated with stigma exsertion and related traits have been identified through genome-wide association analysis using 553 *Oryza sativa* accessions. The combined effect of the grain size genes *GS3* and *GW5* is essential in determining stigma exsertion and its related traits [8].

Plant hormones, which are highly efficient small-molecule organic compounds, play crucial roles in regulating flower development [13, 14]. The increase in auxin (IAA) in tomato line T431 may increase the number of style cells and lead to stigma exsertion, whereas abscisic acid (ABA) has the contrasting effects [5]. The *OsPID*-mediated IAA signaling pathway plays a key role in regulating stigma and ovule formation in rice by maintaining the flower meristem [15]. Gibberellin (GA) induces the expression of flower homologous genes and regulates flower development in *Arabidopsis* [14], as well as controls the elongation and growth of stamens, pistils, and corolla in tomato [16–19]. Cytokinin (CTK) regulates flower number and development by inducing the expression of genes controlling meristem activity in *Arabidopsis* [20]. Jasmonic acid (JA) influences petal cell elongation in *Arabidopsis* [21] and regulates anther cracking, corolla expansion, and nectary development in tobacco [22, 23].

*Nicotiana tabacum* is a heterologous tetraploid self-pollinating plant with high adaptability and genetic stability, which is believed to permanently fix heterozygosity and hybrid vigour [24]. *Nicotiana tabacum* K326, a flue-cured tobacco variety, is widely used in studying fundamental biological processes [25]. Currently, multiple versions of the whole genome sequence of K326 have been published [26], which aids transcriptomic analyses. Male-sterile K326 (MSK326) has been developed through somatic protoplast fusion to improve breeding efficiency. The cytoplasm of MSK326 is derived from *Nicotiana suaveolens*, which is the only sterile cytoplasm determined with no adverse effects on tobacco growth and quality [27]. In addition, we accidentally discovered a stigma exsertion mutant in male-sterile tobacco in the field. F1 hybrids were obtained by crossing mutants (female parents) and cultivated varieties K326 (male parents). Through backcrossing with K326 for eight consecutive generations, a new tobacco line, MSK326SE, with stigma exsertion and cytoplasmic male sterility was obtained [28]. F1 hybrids and future generations showed stable and consistent stigma exsertion and male-sterile traits, demonstrating that stigma exsertion is a cytoplasmic inherited phenotype.

Microscopic observation revealed that the epidermal cell length and width of MSK326SE corolla were significantly decreased compared with that of MSK326 and K326. Further analysis showed that exogenous GA and JA treatment significantly influenced the corolla length, and that the endogenous content of some plant hormones, such as CTK, dihydrozeatinriboside, GA, BR, and JA, was significantly changed in MSK326SE when compared with MSK326 and K326. This indicated that plant hormones play an important role in controlling the corolla shortening of MSK326SE. However, the molecular mechanisms underlying remain unclear.

Here, three homonuclear and heteroplasmic tobacco lines (K326, MSK326, and MSK326SE) were used to explore the mechanism of stigma exsertion in MSK326SE, via transcriptomic methods. Our main objectives were to (1) study the differences in gene expression and plant hormone levels in corolla between stigma exsertion mutants and controls; (2) explore the relationship between stigma exsertion traits, and hormone synthesis and signal transduction; and (3) identify genes involved in regulating stigma exsertion caused by corolla shortening. Our findings will help reveal the molecular mechanism of stigma exsertion in tobacco and further improve the hybrid seed production efficiency of tobacco.

## Results

### Comparative transcriptome profiles between the corollas of different tobacco lines

**Fig. 1:**
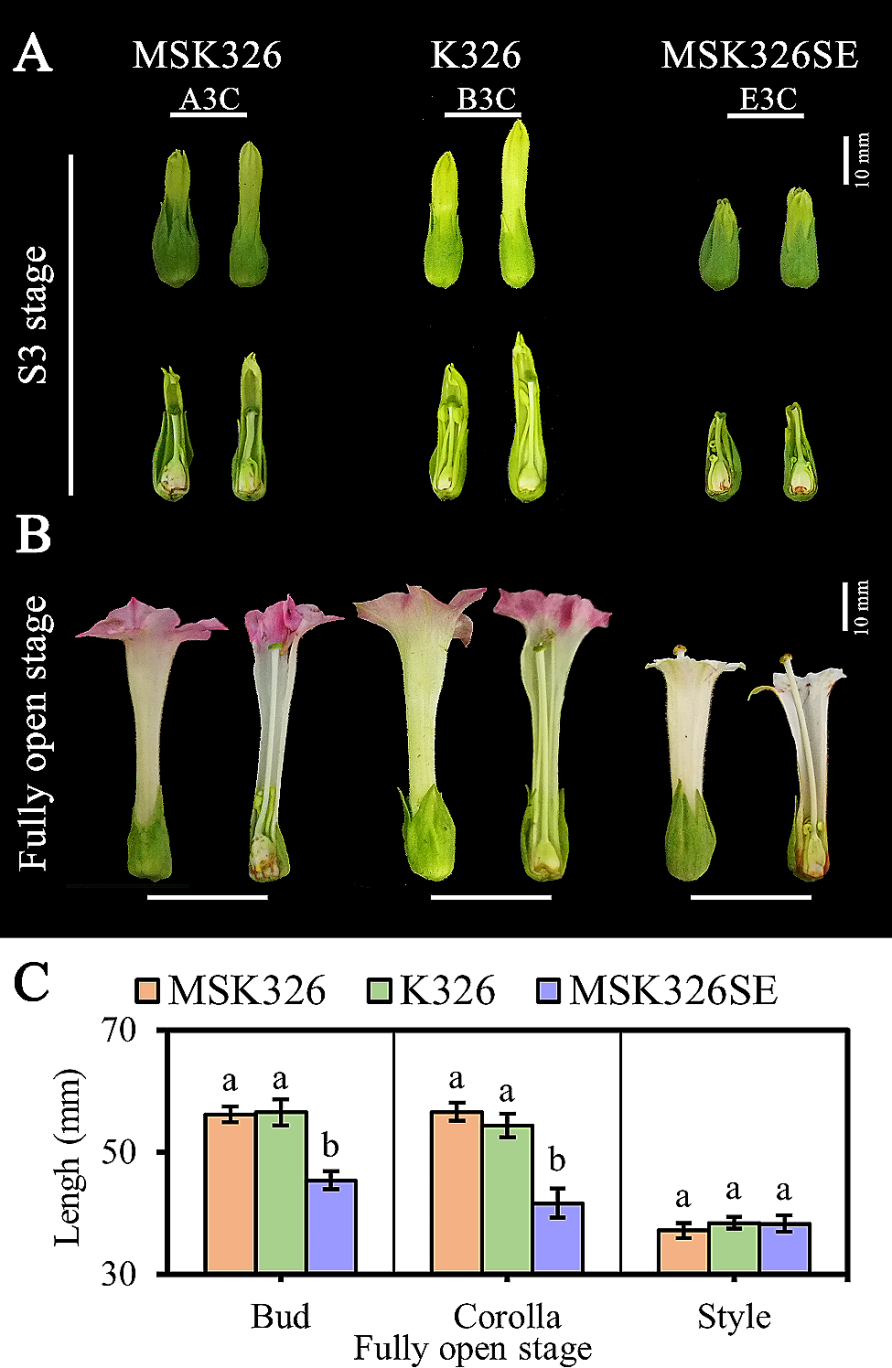
Comparison of flower morphology at different developmental stages. **(A)** Complete and longitudinal sections of flower buds of K326, MSK326, and MSK326SE at the S3 stage. **(B)** Complete and longitudinal sections of flower buds of K326, MSK326, and MSK326SE at the full bloom stage. **(C)** Bud, corolla, and style lengths of K326, MSK326, and MSK326SE at the full bloom stage. Data are the means ± SD of three replicates, with each replicate comprising 20 flowers. Different letters indicate significant differences among the three materials (*P* < 0.05)

Bud, corolla, and style lengths were measured when the flowers of K326, MSK326, and MSK326SE were fully open. The corolla and bud of MSK326SE were significantly shorter than those of MSK326 and K326, whereas the style length was similar among the three lines (Fig. 1B, C), indicating that the shortening of the corolla caused the stigma exsertion of MSK326SE. Our previous study revealed that MSK326SE stigma exsertion began to appear in the S3 stage, when the calyx was fully opened and the corolla started to stretch [28]. Therefore, the corollas of MSK326, K326, and MSK326SE were collected and designated as A3C, B3C, and E3C, respectively, to study the gene expression differences among them (Fig. 1A; Table 1).

**Table 1:** Sample information and code name

Varieties	Character	Tissue	Stage	Sample		
MSK326	Inserted stigma, male sterility, and normal corolla	corolla	S3	A3C-1	A3C-2	A3C-3
K326	Inserted stigma, male fertility, and normal corolla	corolla	S3	B3C-1	B3C-2	B3C-3
MSK326SE	Stigma exsertion, male sterility, and shortened corolla	corolla	S3	E3C-1	E3C-2	E3C-3

**Fig. 2:**
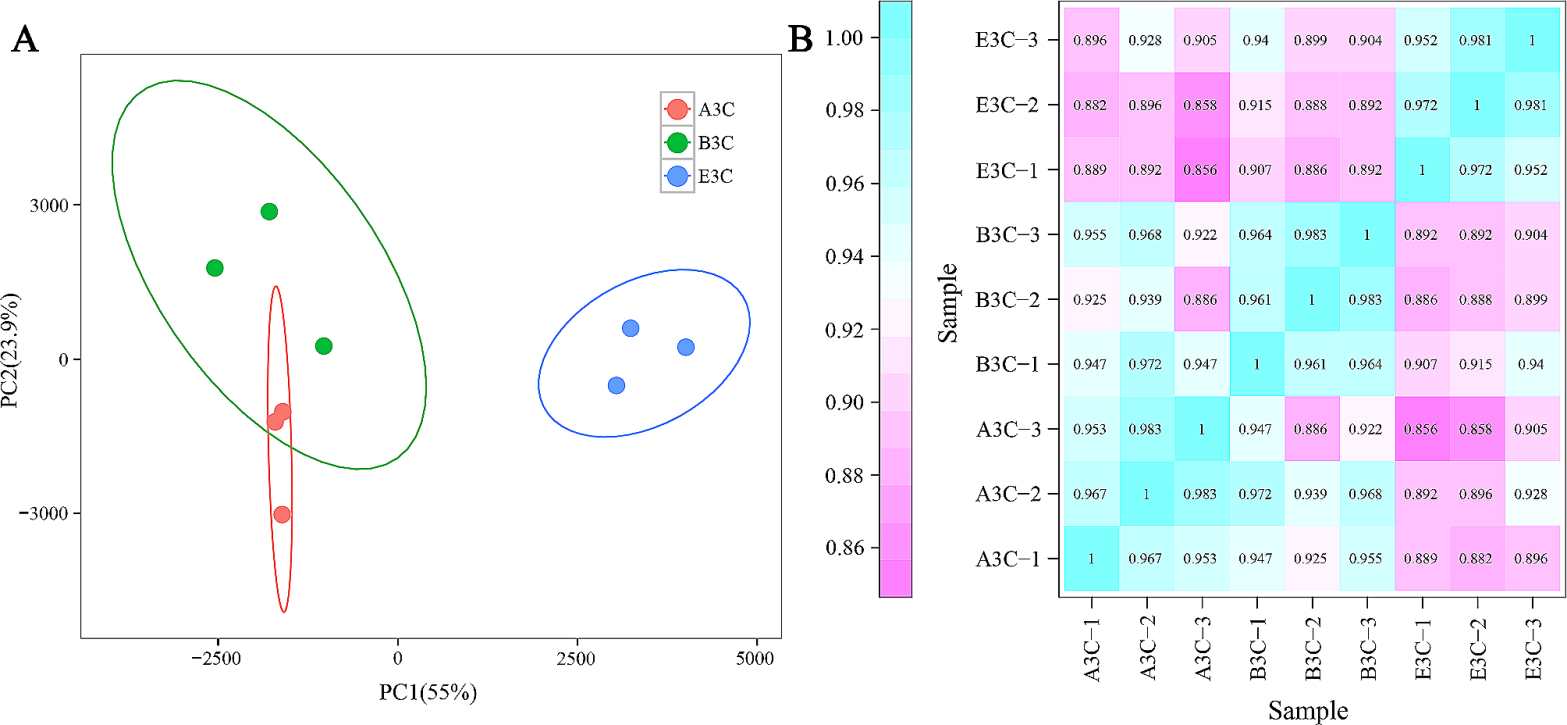
Quality validation of RNA-Seq data. (**A**) Principal component analysis (PCA) plot of different samples. (**B**) Spearman correlation analysis of the different samples

A total of 189,276,837 clean reads were obtained via transcriptome sequencing, with the percentage of Q30 bases ranging from 94.14 to 94.38%. Approximately 90.49–94.92% of the clean reads were mapped to the tobacco reference genome [26] (Table S1). Gene expression patterns of samples from the same group were more highly correlated than those between groups (Fig. 2). These data indicate that the transcriptome quality from the three lines is sufficient for further analysis.

### Candidate gene set that may cause corolla shortening

**Fig. 3:**
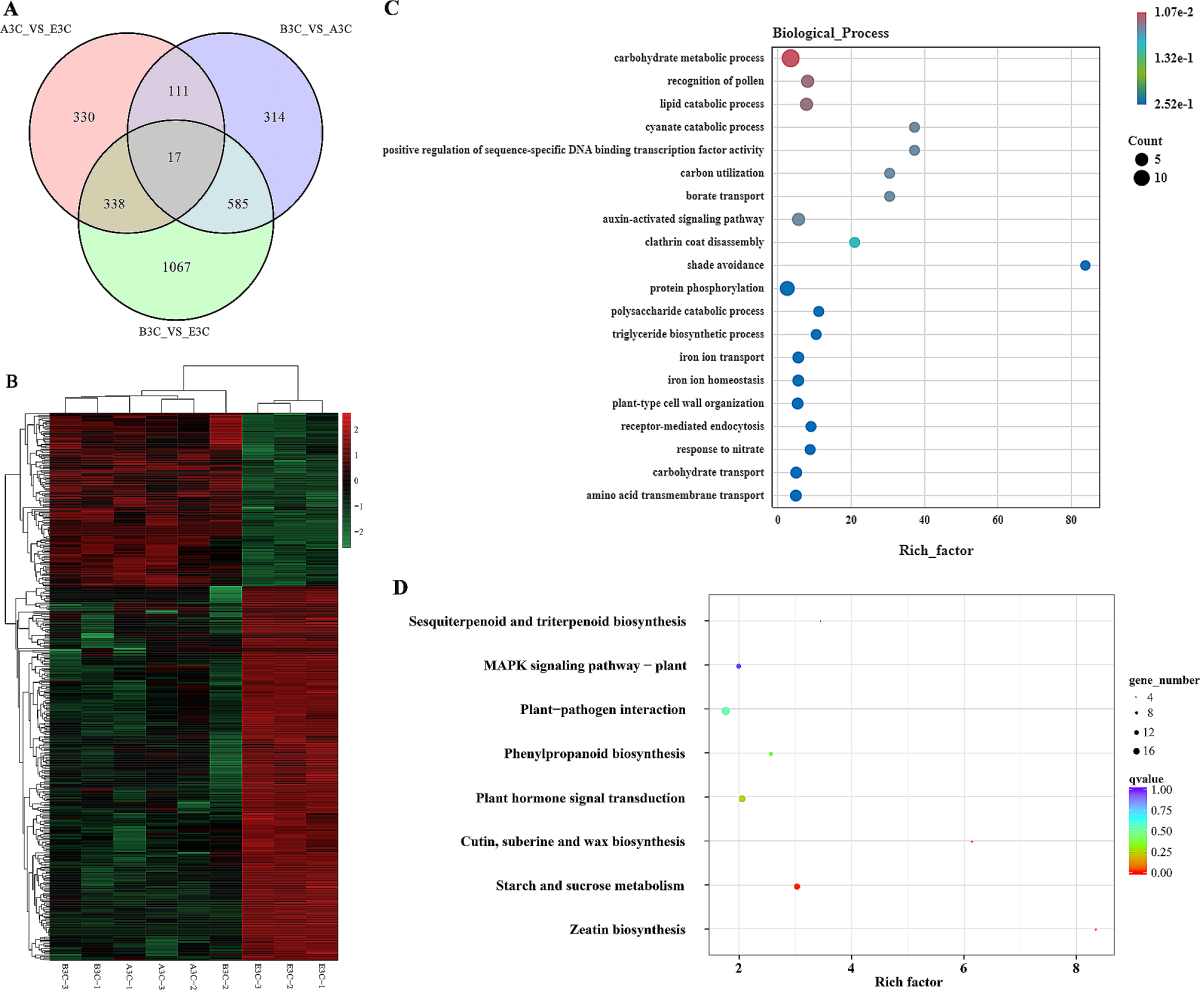
Differentially expressed gene analysis in corolla tissue of K326, MSK326, and MSK326SE. (**A**) Venn diagram of the differentially expressed genes between A3C, B3C, and E3C. (**B**) Expression clustering of the 338 differentially expressed genes in each sample. **(C)** Top 20 most significantly enriched GO terms of the 338 differentially expressed genes. **(D)** Significantly enriched KEGG pathways of the 338 differentially expressed genes

Differentially expressed genes (DEGs) were identified between A3C, B3C, and E3C. 796 genes were differentially expressed between A3C and E3C, 1027 between B3C and A3C, and 2007 between B3C and E3C (Fig. 3A). We speculate that genes equally expressed in A3C and B3C but differentially expressed in E3C correspond to the shortened corolla in MSK326SE. Based on this criterion, we established a candidate gene set containing 338 DEGs (Fig. 3A). The detailed information for the 338 genes is listed in Table S2. Among these genes, 107 were downregulated and 231 were upregulated in E3C (Fig. 3B).

GO enrichment analysis was conducted for the 338 DEGs. The GO terms enriched are listed in Table S3, and the top 20 most significantly enriched GO terms are shown in Fig. 3C. Genes involved in carbohydrate metabolic processes accounted for the highest proportion of the DEGs, followed by genes related to protein phosphorylation, pollen recognition, lipid catabolic processes, and the auxin-activated signaling pathway. The 338 DEGs were also used for KEGG pathway enrichment analysis. Eight pathways were found to be significantly (*P* < 0.05) enriched: sesquiterpenoid and triterpenoid biosynthesis, the MAPK signaling pathway, plant-pathogen interactions, phenylpropanoid biosynthesis, plant hormone signal transduction, cutin, suberine and wax biosynthesis, starch and sucrose metabolism, and zeatin biosynthesis (Fig. 3D).

### Analysis of key DEGs associated with cell division, cell cycle, and expansion

As plant organ size is determined by cell division and expansion, the expression of cell division- and expansion-related DEGs in different samples was investigated. Seven DEGs involved in cell division, cell cycle, or cell expansion were identified. These genes were expressed at similar levels in A3C and B3C but were significantly upregulated or downregulated in E3C. Four genes were significantly downregulated in E3C, including L-ascorbate oxidase homolog (*Bp10.1 and Bp10.2*), *Expansin A11* (*EXPA11*), and *Cyclin A2-2* (*CYCA2-2*). In contrast, *Hypersensitivity related* (*HSR4.1* and *HSR4.2*) and *Meta caspase 1* (*AMC1*) were upregulated in E3C (Table 2). This indicated that these genes may cause changes in both corolla cell division and expansion of the stigma exsertion-mutant.

**Table 2:** Putative differentially expressed genes associated with cell division, cell cycle, and expansion

Gene_ID	FPKM			Annotation
	A3C	B3C	E3C	
gene_10303	18.48	15.52	7.05	L-ascorbate oxidase homolog, Bp10.1
gene_6927	2.95	1.95	0.62	L-ascorbate oxidase homolog, Bp10.2
gene_36421	2.66	2.52	1.03	Expansin-A11-like, EXPA11
gene_36899	4.11	3.44	1.60	Cyclin-A2-2-like, CYCA2-2
gene_21192	0.22	0.18	0.89	Probable mitochondrial chaperone bcs1, HSR4.1
gene_49988	0.68	0.62	2.25	Hyper-sensitivity-related 4-like, HSR4.2
gene_62845	23.48	15.89	60.54	Metacaspase-1-like, AMC1

Note: FPKM values shown here are the average of the three biological replicates

### Analysis of key DEGs involved in endogenous hormone synthesis and signal transduction pathways

**Fig. 4:**
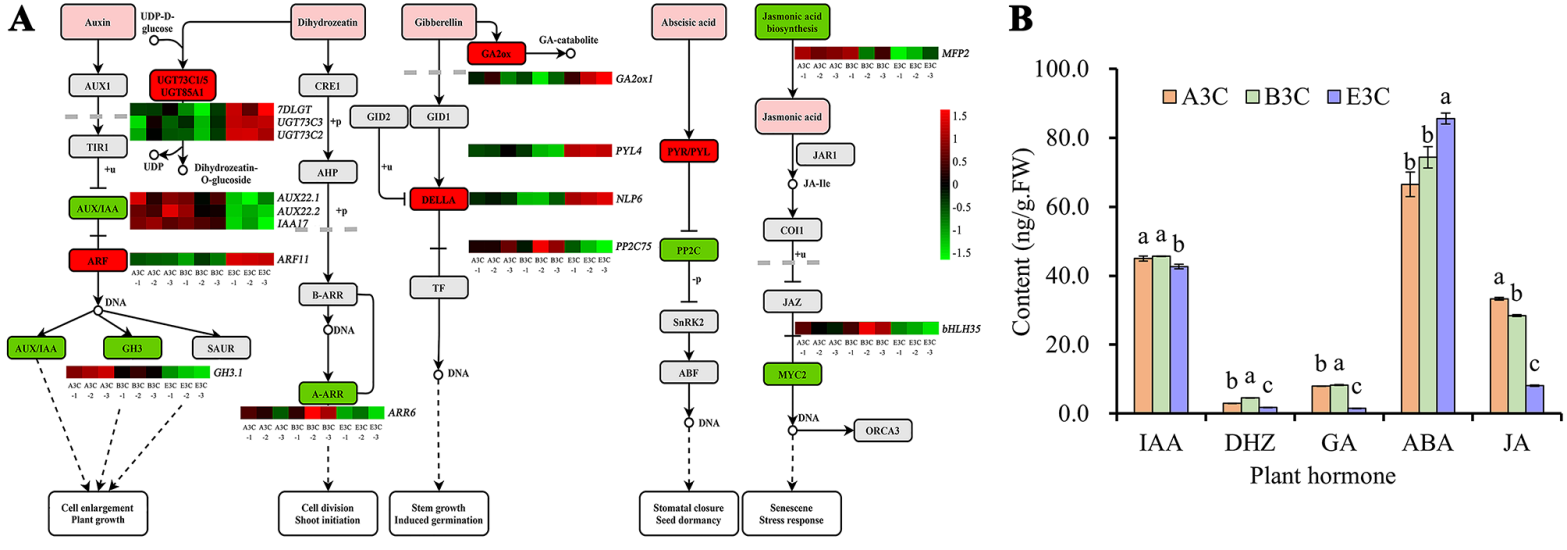
Endogenous hormone synthesis and signal transduction pathways in corolla. **(A)** Heat map of the 15 differentially expressed genes in IAA, zeatin, GA, ABA, and JA synthesis and signal transduction pathways. The red colour of critical enzymes in the KEGG pathway indicates that related genes are upregulated in E3C, and the green colour indicates that related genes are downregulated. **(B)** Hormone levels of IAA, DHZ, GA, ABA, and JA in corolla tissues of the three tobacco lines at the S3 stage

As shown in the KEGG pathway enrichment analysis results, the plant hormone signal transduction pathway was significantly enriched (Fig. 3D). Therefore, we screened the 338 DEGs for those involved in the hormone synthesis and signal transduction pathways, resulting in 15 related DEGs. Among these, five genes were involved in the IAA signaling pathway, with IAA responsive gene (*AUX22.1*, *AUX22.2*, *IAA17*, and *GH3.1*) significantly downregulated in E3C, and *Auxin Response Factor 11* (*ARF11*) significantly upregulated in E3C; four genes were related to the Dihydrozeatin (DHZ) synthesis and signaling pathways, of which the UDP-glucosyltransferase gene (*7DLGT*, *UGT73C3*, *UGT73C2*) were significantly upregulated in E3C, and *Two-component Response Regulator A6* (*ARR6*) was significantly downregulated in E3C; two GA essential genes, *2-beta-dioxygenase 1* (*GA2ox1*) and GA signal transduction repressor DELLA gene (*DELLA*), were found significantly upregulated in E3C; two ABA signaling pathway related genes were identified, the *Pyrabactin Resistance 1-like 4* (*PYL4*) gene was significantly upregulated in E3C, and *Protein Phosphatase 2 C 75* (*PP2C75*) was significantly downregulated in E3C; and finally, two JA signaling pathway genes, *Multi-Function Protein 2* (*MFP2*) and bHLH family gene (*bHLH35*), were significantly downregulated in E3C (Fig. 4A).

Additionally, it was found that the levels of IAA, DHZ, GA, and JA in the corolla of MSK326SE were significantly lower than that of the other two lines, while the ABA level was significantly increased (Fig. 4B).

### Validation of the expression levels of hormone-related genes

**Fig. 5:**
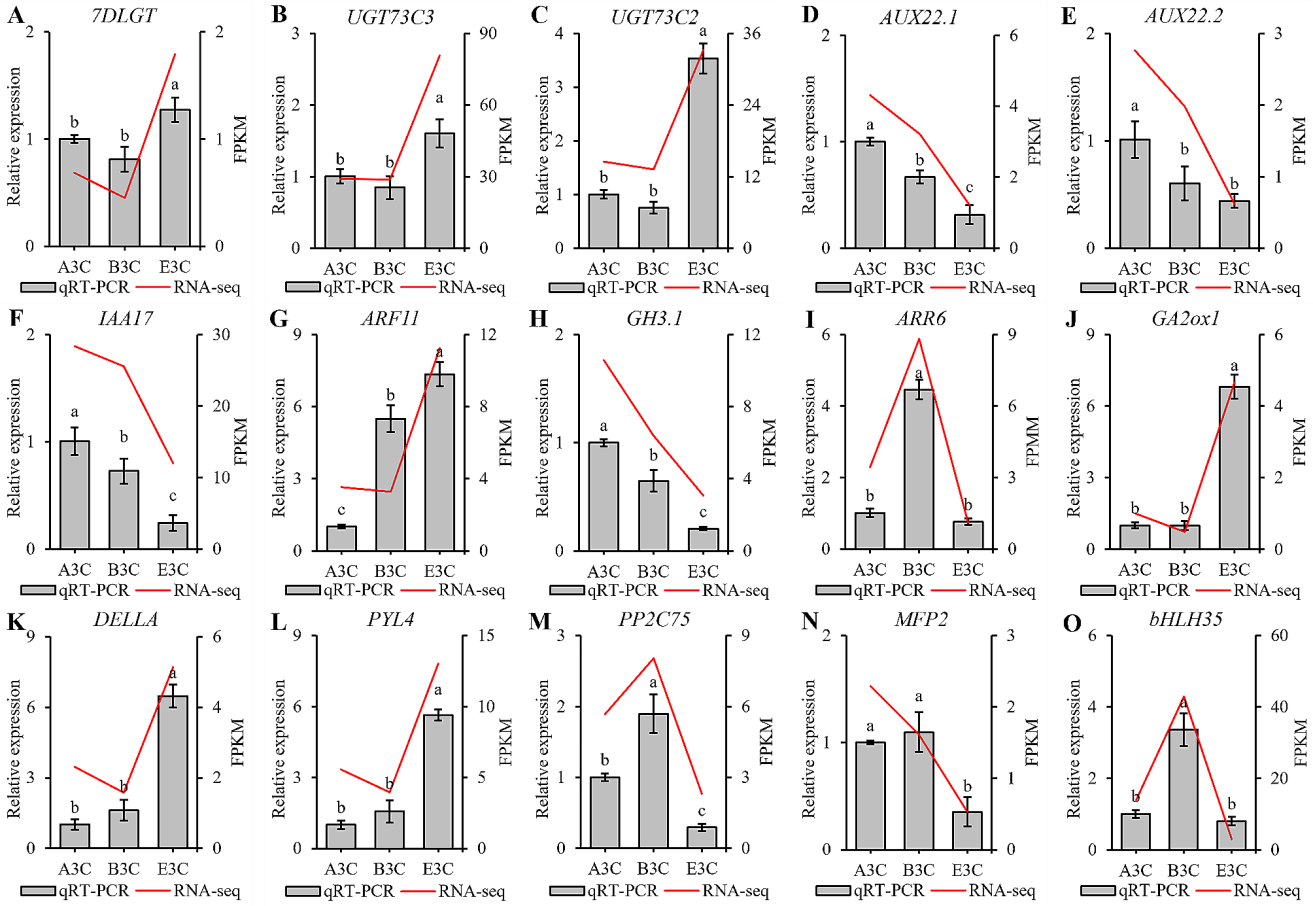
qRT-PCR verification of the relative expression levels of 15 endogenous hormone-related genes in the corolla tissues of the three tobacco lines. The gene expression level of A3C was used as a reference and calculated as 1, and the relative expression level was calculated using the 2^−ΔΔCt^ method. Data are the means ± SD of three replicates. Different letters indicate significant differences among the three lines (*P* < 0.05)

The expression changes of the 15 hormone-related genes mentioned above were validated using quantitative real-time polymerase chain reaction (qRT-PCR). The results showed that the expression levels of *7DLGT*, *UGT73C3*, *UGT73C2*, *ARF11*, *GA2ox1*, D*ELLA*, and *PYL4* were significantly higher in E3C when compared with A3C and B3C, while the expression levels of *AUX22.1*, *AUX22.2*, *IAA17*, *GH3.1*, *PP2C75*, *MFP2*, and *bHLH35* in E3C were lower than that in A3C and B3C. The qRT-PCR data were generally consistent with the transcriptome results, supporting the reliability of the transcriptome sequencing results (Fig. 5).

### Identification and expression profiling of differentially expressed transcription factors (TFs)

**Fig. 6:**
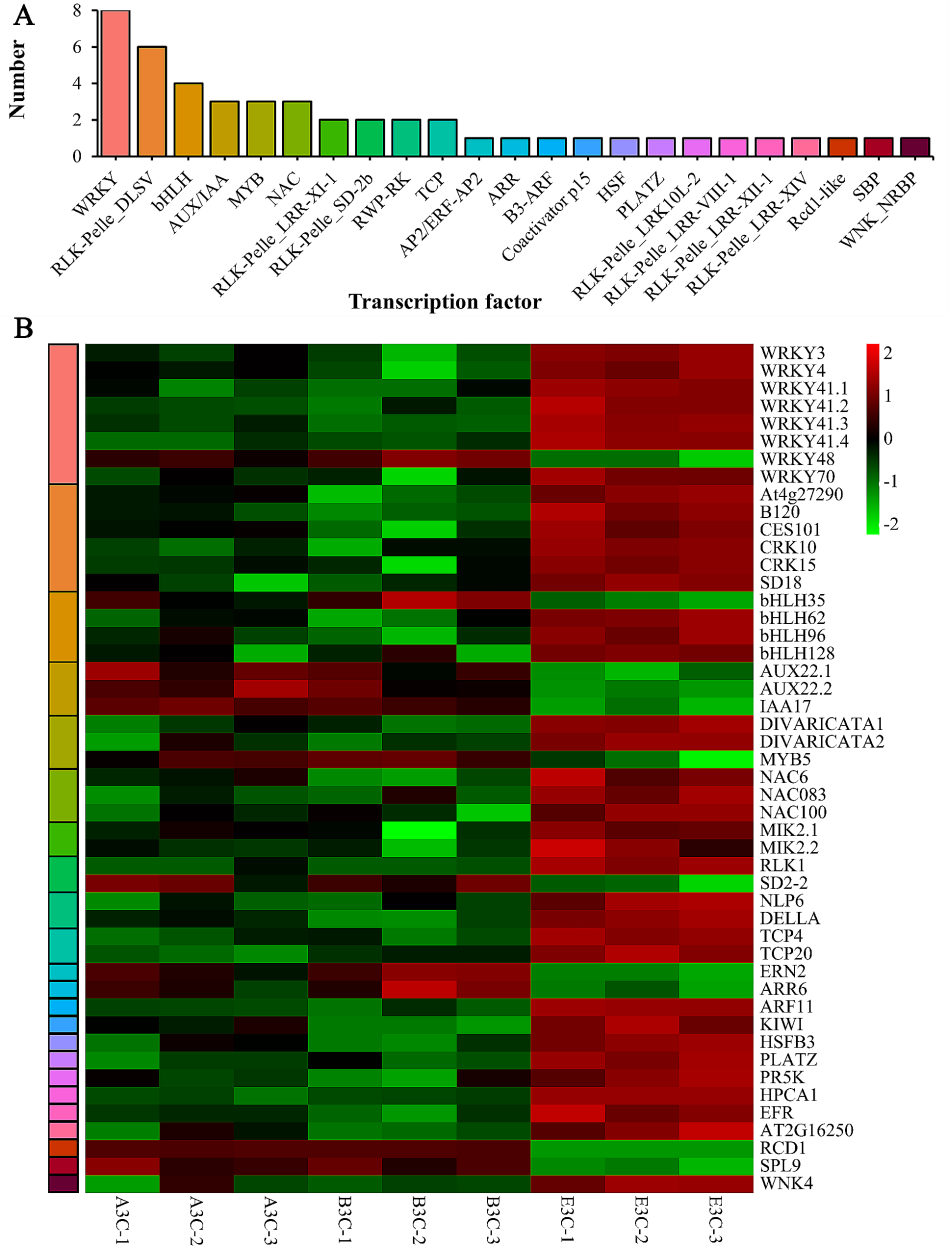
Differentially expressed transcription factors in corolla tissue of K326, MSK326, and MSK326SE. **(A)** Gene numbers of different transcription factor families. **(B)** Heat map of the differentially expressed transcription factor genes among the three tobacco lines

48 TFs were identified from the 338 DEGs. The top three TF families were WRKY, RLK-Pelle_DLSV, and bHLH in terms of gene numbers (Fig. 6A). Notably, twelve TFs were involved in hormone signaling. Among them, six TFs were found to be significantly upregulated in E3C, including those related to IAA (ARF11), BR (MIK2.1, MIK2.2, RLK1, and PR5K), and GA (DELLA) signaling. Conversely, six TFs were significantly downregulated in E3C, related to IAA (AUX22.1, AUX22.2, and IAA17), BR (SPL9), DHZ (ARR6), and JA (bHLH35) signaling (Fig. 6B). The results indicated that hormone signals might be one of the main factors affecting the corolla length of MSK326SE.

### Protein–protein interaction (PPI) network analysis

**Fig. 7:**
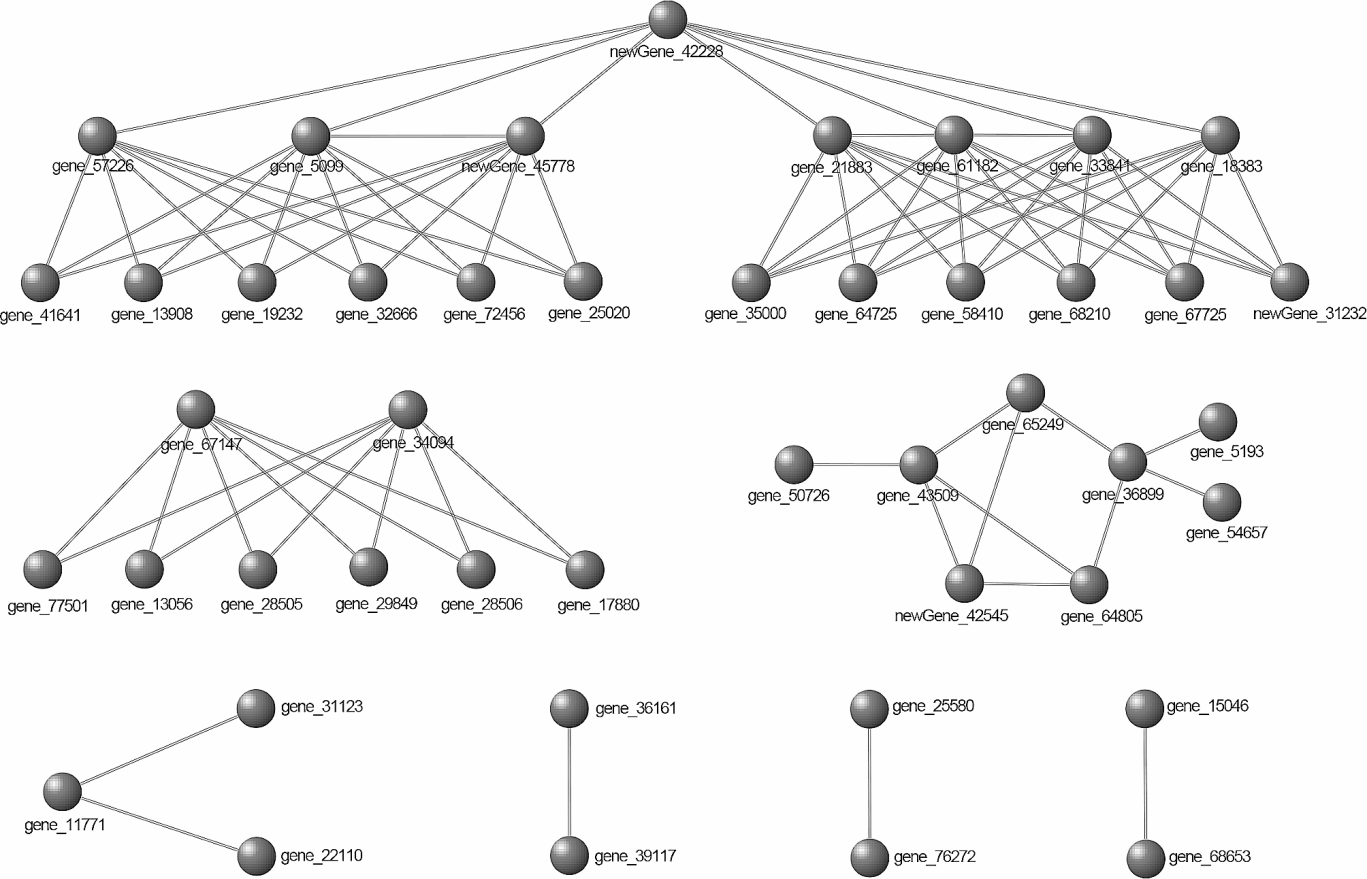
Protein–protein interaction network analysis

The 338 DEGs were subjected to protein-protein interaction network analysis using STRING online webtools [29]. 45 nodes and 79 protein interactions were found by setting FDR < 0.05 and the confident score > 0.9; they could be divided into seven modules (Fig. 7). The largest module contained 20 nodes and 52 protein interactions, and could itself be divided into two parts: one involved in hormone signal transduction, and the other involved in pathogen defensive pathways. The two parts were connected by a protein coded by *newGene_42228*, a serine/threonine-protein kinase, indicating that this may be the hub protein in the network that regulates corolla development in MSK326SE. Other modules were mainly involved in secondary metabolites biosynthesis, mitosis and endocytosis processes, protein translation and proteolysis, transmembrane transport, and DNA reparation.

## Discussion

In tobacco hybrid seed production, because the stigma is enclosed by the corolla, hand pollination inevitably damages the pistil, which affects pollination success and costs labour and time. Interestingly, stigma exsertion helps extend the stigma out of the corolla during flowering, supporting manual pollination, reducing the cost of hybrid seed production considerably, and compensating for the deficiency of hybrid seed production [1, 2]. In our previous study, we found a stigma exsertion line, MSK326SE [28]. However, the mechanism underlying stigma exsertion in this tobacco line has not yet been elucidated. Therefore, in the present study, transcriptome analysis and hormone measurements were performed to identify the essential genes and hormone metabolic pathways that regulate stigma exsertion caused by corolla shortening and to provide new insights into the mechanism of stigma exsertion in tobacco.

Plant hormones play an essential role in flower growth and development, with varying effects among different floral tissues [30]. Based on the results obtained in this study, we hypothesize that plant hormones also play an important role in the corolla shortening of MSK326SE.

The IAA has been shown to positively regulate corolla development [31]. In our RNA-Seq analysis, we found that the expression of four IAA-induced genes (*AUX22.1*, *AUX22.2*, *IAA17* and *GH3.1*) was downregulated, these genes are responsible for IAA signaling and homeostasis [32, 33]. Consistent with these results, the levels of IAA in the corolla of MSK326SE were significantly reduced, which likely inhibited corolla cell enlargement and growth.

CTKs play vital roles in cell division [34] and are known to promote petal development [35]. It has been shown that in the zeatin biosynthesis pathway, DHZ (with CTK activity) could be glycosylated to dihydrozeatin-O-glucoside (without CTK activity), under the catalyse of UDP-glucosyltransferases [36]. In this study, we found that three UDP-glucosyltransferase genes, *7DLGT*, *UGT73C3*, and *UGT73C2*, were upregulated in MSK326SE. What’s more, the expression of the *A-ARR* gene (*ARR6*) [37] was downregulated. Therefore, we speculate that the transmission of the zeatin signal in the corolla of MSK326SE may be obstructed, eventually affecting cell division and corolla development.

GAs are known to promote flower development, and in *Arabidopsis*, GA-deficient mutants exhibit impaired petal development [38]. DELLA is an essential regulatory protein of the GA signal transduction pathway. At low GA levels, the DELLA protein can bind to and block the activity of transcription factors affecting plant growth [39, 40]. We found that the expression level of *DELLA* was significantly upregulated in the MSK326SE corolla. In addition, given that the GA2-oxidases (GA2oxs) can turn GA into its inactive form [41], we found that the expression of *GA2ox1* was also significantly upregulated in MSK326SE corolla, this might block the GA signal transduction.

ABA is considered an inhibitor of plant growth and development [42], and exogenous application of ABA has been shown to significantly suppress petal development in *Gerbera hybrida* [43]. We found that ABA levels in the MSK326SE corolla were increased. In the ABA signaling pathway, the binding of ABA to its receptor protein PYR/PYL inhibits the activity of type 2 C protein phosphatase (PP2C), thereby initiating the expression of a series of ABA-induced genes [44]. In this study, we found that the expression of the ABA signal receptor gene *PYR/PYL* was upregulated, while the expression of type 2 C protein phosphatase coding gene *PP2C75* was downregulated in MSK326SE, which likely promote ABA signal transmission and inhibit petal development.

JAs play a vital role in flower development. In tomato, JA is involved in regulating the division and expansion of the stamen and pistil cells, resulting in stigma exsertion [6]. We found that the expression of the JA synthesis gene *MFP2* and the MYC2 transcription factor gene *bHLH35* was downregulated in the MSK326SE corolla, which likely disrupted JA signal transduction and ultimately influenced corolla development.

Phytohormones regulate floral organ development through a complex network and crosstalk, rather than through isolated pathways [45]. In the *Arabidopsis* flower, both GA and IAA can promote the biosynthesis of JA [46, 47], suggesting that the decrease in JA content in MSK326SE corolla found in this study may be partially due to a decrease in GA and IAA content. Additionally, a study in *Gerbera hybrida* found that a zinc finger protein could mediate the crosstalk between GA and IAA during flower development [48]. Thus, we propose that GA, IAA, and JA may work synergistically to regulate corolla shortening in MSK326SE.

ABA may also influence the function of other hormones. A previous study found that ABA could upregulate the expression of *GA2ox1* [49]. Consistent with this, we found that ABA content was increased while GA content was decreased in MSK326SE. Antagonistic effects of ABA against IAA [5], CTKs [50, 51], and GA [43] in regulating flower development have also been reported. This suggests that ABA may inhibit corolla development in MSK326SE by suppressing the effects of IAA, CTKs, and GA.

In the PPI network analysis, we found that the largest module contained more than half of the protein interactions; interestingly, this module was connected by a serine/threonine-protein kinase (Fig. 7). According to the KEGG enrichment analysis, this protein was involved in the MAPK signaling pathway (Fig. 3D). Previous studies have shown that MAPK cascades play an important role in regulating floral organ development, such as the ovule [52] and pollen [53]. Additionally, MAPK pathway components are also functionally associated with the biosynthesis and signaling of plant hormones such as auxin, ethylene, JA, ABA, and SA [54]. Thus, we speculate that the serine/threonine-protein kinase identified in this study may work as the hub protein to integrate the signals of the different hormones, eventually leading to corolla shortening. Further studies are needed to verify the function of this protein.

## Conclusions

The regulatory mechanisms underlying corolla shortening leading to stigma exsertion in tobacco were analyzed by combining corolla transcriptome and endogenous hormone level data. The levels of IAA, DHZ, GA, and JA in the corolla of tobacco with stigma exsertion decreased significantly, whereas the levels of ABA increased significantly. 15 critical genes in the hormone synthesis and signal transduction pathways were differentially expressed. In addition, a putative hub gene coding a serine/threonine-protein kinase were identified. Our findings provide important information for the further molecular verification of stigma exsertion genes in *N. tabacum*.

## Materials and methods

### Plant materials and growth conditions

Three homonuclear-heteroplasmic tobacco lines, MSK326SE with stigma exsertion and male sterility, MSK326 with inserted stigmas and male sterility, and K326 with inserted stigmas and male fertility, were used as the study materials. More detailed information about the three lines has been described in our previous study [28].

The three lines were planted in a field at the Xuchang experimental station of Henan Agricultural University, China. All lines were transplanted on 11 May 2021 with a row spacing of 120 cm and a plant spacing of 50 cm. Samples were collected on 21 August 2021 after the flowering stage commenced. During this stage, the average temperature ranged from 20.0 to 30.3 °C, with a monthly average precipitation of 110.3 mm and sunshine duration of 14.0 h.

Our previous study found that the significant corolla shortening of tobacco MSK326SE was the decisive factor for its stigma exsertion. We also divided the flower buds into five developmental phases according to the morphology of the flower organs [28]. In the current study, corolla tissues from the three lines were collected at the S3 stage in which the calyx of the three materials was fully opened, the corolla began to stretch, and the stigma of MSK326SE began to exsert the corolla (Fig. 1A). Samples were immediately frozen in liquid nitrogen and stored at − 80 °C for subsequent transcriptome sequencing analysis. Three replicates were used for all materials, and each replicate comprised 20 corollas (Table 1).

### RNA-Seq library preparation and sequencing

Total RNA was extracted from the corolla using TRIzol reagent (TaKaRa, Japan). RNA concentration and purity were measured using a NanoDrop 2000 spectrophotometer (Thermo Fisher Scientific, USA). RNA integrity was assessed using the RNA Nano 6000 Assay Kit on the Agilent Bioanalyzer 2100 system (Agilent Technologies, USA). The extracted corollas total RNA was sent to Biomarker Technologies Co., Ltd. (Beijing, China) for library construction, followed by sequencing using the Illumina NovaSeq6000 platform with a paired-end sequencing read length of 150 bp.

### RNA-Seq data analysis

The raw reads were further processed using the bioinformatic pipeline tool, BMKCloud (http://en.biocloud.net/). Clean reads were obtained by removing reads containing adapters, poly-N, and low-quality reads from the raw data, and then mapped to the *N. tabacum* L. cv. K326 reference genome [26] using Hisat2 tools [55].

Gene function was annotated using the following databases: NCBI non-redundant protein sequences (NR) and non-redundant nucleotide sequences (NT) database [56], protein family (Pfam) [57], Clusters of Orthologous Groups of proteins (KOG/COG) [58], Swiss-Prot (a manually annotated and reviewed protein sequence database) [59], Kyoto Encyclopedia of Genes and Genomes (KEGG Ortholog database) [60], Gene Ontology (GO) [61], and evolutionary genealogy of genes: Non-supervised Orthologous Groups (eggNOG) [62].

Gene expression levels were quantified based on the fragments per kilobase of transcript per million mapped fragments (FPKM). DEGs were identified using DESeq2 software [63] by setting the adjusted *P*-value < 0.01 and the fold change ≥ 2 as the thresholds.

### Identification of differentially expressed TFs

All the amino acid sequences of the DEGs were searched against the Plant Transcription Factor Database v5.0 (PlantTFDB, http://planttfdb.gao-lab.org) using the BLAST tool with an *E*-value cut-off of 1.0×*E*^− 5^.

### GO and KEGG enrichment analysis of DEGs

GO enrichment analysis of the DEGs was performed using the GOseq R package [64]. The KOBAS software [65] was used to test the statistical enrichment of the DEGs in the KEGG pathways.

### Determination of endogenous hormone levels

To determine the IAA, DHZ, GA, ABA, and JA levels, 0.5 g corolla tissue was collected at the S3 stage from each plant, frozen in liquid nitrogen, and stored at − 80 °C. Hormone content was analysed using a double antibody one-step sandwich enzyme-linked immunosorbent assay (ELISA) [66]. Three biological replicates were used for each line.

### Validation of gene expression using qRT-PCR

For each line, three biological replicates were used, and three technical replicates were set for each biological replicate. qRT-PCR was performed using SYBR Green PCR Master Mix (Beijing Conway Biotechnology Co., Ltd., China) in a StepOnePlus instrument (Applied Biosystems, USA). The PCR mixture components were set according to a previous study [67]. The cycling condition was as follows: pre-denaturation at 95 °C for 3 min, then 40 cycles at 94 °C for 10 s (denaturation), 58 °C for 20 s (annealing), and 72 °C for 20 s (extension). The 2^−ΔΔCt^ method [68] was used to calculate the relative expression levels. The primer sequences used for qRT-PCR are listed in Table S4.

### Statistical analysis

All data were obtained from three biological replicates and were expressed as mean ± standard deviation (SD). Data obtained were subjected to one-way analysis of variance (ANOVA) using the Statistical Product Service Solutions (SPSS) software (v. 26.0, IBM, New York, USA), and significance was tested using Duncan’s test; *P* < 0.05 was considered statistically significant.

### Electronic supplementary material

Below is the link to the electronic supplementary material.

Supplementary Material 1

Supplementary Material 2

Supplementary Material 3

Supplementary Material 4

## Data Availability

The transcriptome clean raw reads data that support the findings of this study have been submitted to NCBI Sequence Read Archive (SRA) under Accession (SAMN28772225 - SAMN28772233), Bioproject: PRJNA843899. All data generated or analysed during this study are included in this published article and its supplementary information files. The authors are pleased to share the data upon request.

## Abbreviations

ABA: Abscisic acid
ARF: Auxin response factor
CTK: Cytokinin
DEG: Differentially expressed gene
DHZ: Dihydrozeatin
FPKM: Fragments per kilobase of transcript per million mapped fragments
GA: Gibberellin
GO: Gene Ontology
IAA: Auxin
JA: Jasmonic acid
KEGG: Kyoto encyclopedia of genes and genomes
PPI: Protein–protein interaction
qRT-PCR: Quantitative real time polymerase chain reaction
QTL: Quantitative trait loci
