# Supplementary material for: Transcriptome disclosure of hormones inducing stigma exsertion in Nicotiana tabacum by corolla shortening
Source: BMC Genomics. 2024 Mar 28;25:320. doi: 10.1186/s12864-024-10195-4 (PMC10976690; doi:10.1186/s12864-024-10195-4)
Supplement: Supplementary file 1 — Supplementary Material 1 [file 12864_2024_10195_MOESM1_ESM.docx]

**Table S1.** Transcriptome sequencing data statistics and comparison efficiency with the reference genome.

| **Samples** | **Clean reads** | **Clean bases** | **GC (%)** | **Q30** | **Mapped Reads** |
| --- | --- | --- | --- | --- | --- |
| A3C-1 | 19,648,412 | 5,856,171,444 | 43.04% | 94.19% | 90.49% |
| A3C-2 | 19,387,262 | 5,785,402,036 | 43.03% | 94.38% | 91.72% |
| A3C-3 | 20,881,846 | 6,231,578,676 | 43.09% | 94.26% | 90.90% |
| B3C-1 | 21,282,281 | 6,353,688,032 | 43.00% | 94.14% | 93.62% |
| B3C-2 | 20,549,350 | 6,133,329,282 | 42.94% | 94.23% | 94.92% |
| B3C-3 | 20,594,370 | 6,143,094,542 | 43.04% | 94.31% | 94.23% |
| E3C-1 | 23,235,843 | 6,932,609,750 | 42.93% | 94.38% | 92.14% |
| E3C-2 | 21,462,542 | 6,389,770,312 | 42.86% | 94.30% | 93.42% |
| E3C-3 | 22,234,931 | 6,629,444,932 | 42.74% | 94.18% | 92.96% |
