# Supplementary material for: Transcriptome disclosure of hormones inducing stigma exsertion in Nicotiana tabacum by corolla shortening
Source: BMC Genomics. 2024 Mar 28;25:320. doi: 10.1186/s12864-024-10195-4 (PMC10976690; doi:10.1186/s12864-024-10195-4)
Supplement: Supplementary file 4 — Supplementary Material 4 [file 12864_2024_10195_MOESM4_ESM.docx]

**Table S4.** Primer sequences of genes used for quantitative real-time PCR.

| **Primer name** | **Primer sequences (5'-3')** | **Related protein** |
| --- | --- | --- |
| *NtL25*-F | CCTCACCACAGAGTCTGCAA |  |
| *NtL25*-R | GTTGGCAACGTCCAAAGCAT |  |
| *7DLGT*-F | TCTGATGGGAAAAGAAGACCAGT | UGT73C1/5  UGT85A1 |
| *7DLGT*-R | GGCTCATCCAAGTCATCACCTTT |  |
| *UGT73C3*-F | AGCAGTGTTGAGGCAAGTTCTTT |  |
| *UGT73C3*-R | AGAAACTGAGAAATGAAAACACAGC |  |
| *UGT73C2*-F | AGAGGATGGGCTCCTCAAGTAT |  |
| *UGT73C2*-R | GTAGTCCCGTTGAAATACCTTCC |  |
| *AUX22.1*-F | AGATGTTCCATGGCAAATGTTCA | Aux/IAA |
| *AUX22.1*-R | ACTAGGCCAATGATAAGACAGGA |  |
| *AUX22.2*-F | CGATGTTCCATGGCAAATGTTC |  |
| *AUX22.2*-R | ACTAGGCCAGTGGTAACTGAT |  |
| *IAA17*-F | TCGACCCTTCCCCTTTCGAG |  |
| *IAA17*-R | CAATATGGCCTGCCTTGACC |  |
| *ARF11*-F | ACACACTCACACACGCAAGT | ARF |
| *ARF11*-R | ATTCCCTATACAGATCATCATCACC |  |
| *GH3.1*-F | AGCTTCGACAGTTTCCATTAGC | GH3 |
| *GH3.1*-R | TACAAACAGATTGGAGGGGCA |  |
| *ARR6*-F | GCTCGCATTGATAGATGTTTGG | A-ARR |
| *ARR6*-R | CCCTCGCCCCTCAGTATAAAA |  |
| *GA2ox1*-F | AGCCATTGAGCACTTGTCCA | GA2ox |
| *GA2ox1*-R | GGACGCTATGGTTGACGACT |  |
| *DELLA*-F | CTCCGCAGAATGGCTTTCTATATG | DELLA |
| *DELLA*-R | CAGAGAGCCTCCCGCTTT |  |
| *PYL4-F* | CATCGTAGGGGATGGTGACG | PYR/PYL |
| *PYL4-R* | GGTGGTGACGGAACGGTAAT |  |
| *PP2C75*-F | TCCAAAACCATTCTCCGCCT | PP2C |
| *PP2C75*-R | CTCGTGAAGGATTGACCCGAA |  |
| *MFP2*-F | TGGGCAGGGATGAGCTACTT | MFP2 |
| *MFP2*-R | CATCCTACGTGGTGTCCTCA |  |
| *bHLH35*-F | AGCATAACGTGTCTGCTTCCT | MYC2 |
| bHLH35-R | GCCGAGATTGCCTCATCGAA |  |
